# Phytohormone Profiling Method for Rice: Effects of *GA20ox* Mutation on the Gibberellin Content of Japonica Rice Varieties

**DOI:** 10.3389/fpls.2019.00733

**Published:** 2019-06-07

**Authors:** Camilo López-Cristoffanini, Xavier Serrat, Olga Jáuregui, Salvador Nogués, Marta López-Carbonell

**Affiliations:** ^1^Departament de Biologia Evolutiva, Ecologia I Ciències Ambientals, Secció de Fisiologia Vegetal, Universitat de Barcelona, Barcelona, Spain; ^2^Scientific and Technological Centers, Universitat de Barcelona, Barcelona, Spain

**Keywords:** *GA20ox* gene, gibberellins (GA), Mediterranean japonica rice, phytohormone analysis, dwarfism, HPLC-MS/MS, solid phase extraction

## Abstract

Gibberellins (GAs) are a very important group of phytohormones involved in seed germination, vegetative growth, flowering, and fruit development, being only 4 of the 136 known bioactives: GA_1_, GA_3_, GA_4_, and GA_7_. It has been evidenced that mutations in the *OsGA20ox-2* gene produce rice (*Oryza sativa*) dwarf varieties, which were one of the main pillars of the green revolution. In this work two main objectives were proposed: (i) develop a rapid and broad phytohormone profiling method and (ii) to study the effects on the GA content of the *GA20ox-2* mutation in several rice developmental stages using three varieties (tall variety, elite variety, mutated variety). A phytohormone extraction using an SPE step and HPLC-MS/MS detection using a QqQ instrument was determined which resulted in limits of detection (LOD) and limits of quantification (LOQ) for GAs that varied between 0.1–0.7 and 0.3–2.3 pg ⋅ g^-1^ (f.w.) of rice sample, respectively, allowing highly sensitive phytohormones detection in samples. Moreover, a good reproducibility was obtained for the GAs as relative standard deviations (RSD) for a 40 ng ⋅ mL^-1^ pattern varied between 0.3 and 0.9%. Notoriously, GA_1_ was absent in the coleoptile and GA_4_ was the GA with higher content in the majority of developmental stages. We also observed a large content increase of the four bioactive GAs in the internode of the flag leaf of the mutated variety allowing to reach same height as the elite variety. Therefore, we provide a rapid and broad phytohormonal profiling method and evidence that the *GA20ox-2* mutation is not the only factor generating dwarf varieties. To our knowledge, this is the first study that it has been reported such a high number of simultaneously analyzed gibberellins in rice samples (*Oryza sativa* ssp. japonica) in different tissues of different growth stages.

## Introduction

Plants rely on plant hormones, also called phytohormones, for several processes throughout their life including growth, development and responses to stress. These small molecules are naturally occurring substances that act at very low concentrations and have signaling functions (Davies, 2010; Kudo et al., 2013). Nowadays, there is large knowledge regarding phytohormone biosynthesis, regulation and their specific role in signaling (Peleg and Blumwald, 2011). Gibberellins, a large hormone category, are a large group of tetracyclic diterpenoid carboxylic acids, which were first identified as secondary metabolites of the fungus *Gibberella fujikuroi* (Hedden and Thomas, 2012). Nowadays, more than 136 different gibberellin structures have been found, but four of them are highly bioactive: GA_1_, GA_3_, GA_4_, and GA_7_ (Hedden and Phillips, 2000; Yamaguchi, 2008; Macías et al., 2014). Moreover, these four bioactive GAs have been detected in different rice developmental stages (Ma et al., 2011; Wu et al., 2012).

Gibberellin biosynthesis, in plants, begins in plastids where *trans*-geranylgeranyl diphosphate (GGPP) is converted in two steps to *ent*-kaurene. Then, this molecule goes to the endoplasmic reticulum where it is converted into gibberellin GA_12_ where it can follow two pathways: (i) the GA_12_- or non-hydroxylated gibberellins pathway or (ii) be synthesized to GA_53_ through the GA 13-oxidase (*GA13ox*) to follow the GA_53_- or hydroxylated gibberellin pathway (Yamaguchi, 2008; Urbanová et al., 2013). Interestingly, both pathways have the same enzymes where GA 20-oxidase (*GA20ox*) produces GA_9_ and GA_20_ for GA_12_- and GA_53_- pathways, respectively (Yamaguchi, 2008). Then, by the action of GA 3-oxidase (*GA3ox*) the bioactive gibberellins are produced: GA_1_ and GA_3_ (GA_53_-pathway) and GA_4_ and GA_7_ (GA_12_-pathway) (Hedden and Phillips, 2000). In addition, the GA 2-oxidases (*GA2oxs*) are enzymes that deactivate gibberellins through a change of the -OH position (Hedden, 2001). All these findings have revealed that there are several steps for GAs biosynthesis regulation including genes for activation/deactivation and phytohormones interaction at several levels of the biosynthesis pathways (Wang et al., 2017).

Gibberellins have been largely viewed as phytohormones involved in processes such as seed germination, vegetative growth, flowering, and fruit development (Olszewski et al., 2002); but their main focus since 1960 (during the green revolution) has been their involvement in dwarfism traits of plants (Hedden, 2003). This reduction in height allowed to obtain high-yielding varieties which had a significant change in GA biosynthesis and signaling pathway (Hedden, 2003; Wang et al., 2017). Nevertheless, some semi-dwarf or dwarf mutants defective in hormone biosynthesis or signaling have undesirable secondary effects such as altered tillering, small grains, semi-sterility, malformed panicles and lower plant establishment (Liu F. et al., 2018). In rice, four different mutations in the GA_20_ oxidase 2 gene (*GA20ox-2*, which has three exons) have been found to provoke a disruption in GA biosynthesis which generates plants with dwarfism traits, named *sd-1* mutants (Sasaki et al., 2002). For indica varieties, the mutation is generally a 383-bp deletion (Dee-geo-woo-gen, between exon 1 and 2), whereas for japonica varieties the mutations are point mutations (Jikkoku in exon 1, Calrose in exon 2, and Reimein in exon 3) that result in single amino acid substitutions (Sasaki et al., 2002; Hedden, 2003). Independently of the allele that provokes height reduction, the gibberellins production pattern is disrupted as *sd-1* mutated plants show GA_53_ accumulation and a lower content of the gibberellins that are produced by *GA20ox-2* (Ashikari et al., 2002; Sasaki et al., 2002; Spielmeyer et al., 2002). Moreover, these mutants are only defective in GA biosynthesis, and not in GA perception, as external GA applications allow to recover normal height (Hedden, 2003). In addition to their crucial role in regulating plant height, they have also been shown to be involved in tolerance to abiotic stress such as salinity which can severely affect yield (Iqbal et al., 2012; Reddy et al., 2017).

Gibberellins are present in plants at very low concentrations that can range between 0.9 and 16.8 ng ⋅ g^-1^ of fresh weight, hence GAs in samples should be enriched prior to detection (Chen et al., 2012). Crucial points during gibberellin analysis are extraction and cleaning steps, which should ensure high presence of GAs and low presence of other molecules (Urbanová et al., 2013). The majority of current GAs extraction methods use the classic liquid-liquid extraction and solid phase extraction (SPE) with reverse phase C-18 cartridge for sample concentration and clean up (Macías et al., 2014). Nowadays, HPLC-MS/MS is the standard and routine technique for GAs separation and detection (Urbanová et al., 2013; Macías et al., 2014) mainly using triple quadrupole instruments for their quantification at trace levels. The biggest problem in plants, including rice, is the difficulty to detect a high number of gibberellins in one run (Kojima et al., 2009; Chen et al., 2012; Urbanová et al., 2013).

Due to the crucial role of gibberellins in regulating process such as growth, development and abiotic stress, and thanks to the current advances in HPLC-MS/MS techniques there is increased interest in studying whole hormonal profiles. Therefore, we report, for the first time, the application of a rapid and broad phytohormone profiling method, with high specific and accuracy, that can detect in one single run a total of 16 phytohormones (including 13 different gibberellins) on rice samples from different tissues and reproductive stages (*Oryza sativa*), using an SPE step and HPLC-MS/MS detection. This method was used to study the effect of a *GA20ox* mutation in three Mediterranean japonica rice varieties with differential heights: NRVC980385 [Ebro Delta elite variety, Serrat et al. (2014)], Bomba [Ebro Delta traditional variety, Franquet Bernis and Borràs Pàmies (2004)] and *dwarf*-Bomba (traditional variety with phenotypical dwarfism traits, field observations).

## Materials and Methods

### Plant Material and Sampling

Three Mediterranean japonica rice varieties were used in this study: NRVC980385 (N), Bomba (B), and *dwarf*-Bomba (*d*B). The dwarfism trait of *dwarf*-Bomba was verified by PCR and posterior Sanger sequencing. For this, DNA of the three varieties was extracted according to Doyle and Doyle (1987) with slightly modifications. Exon 2 of the *GA20ox-2* gene was amplified using the primers designed by Spielmeyer et al. (2002) following the PAQ5000 (Agilent, Santa Clara, CA, United States) manufacturer instructions. Afterward, the PCR product was sequenced using Sanger method by the Genomic platform of the CCiT-UB (Barcelona, Spain).

Plants were germinated in Petri dishes with a humid autoclaved paper and in addition grown in greenhouse conditions at the Experimental Fields Service at the University of Barcelona (Barcelona, Spain) on four-liter plastic containers filled with rice substrate as described in Serrat et al. (2014). For greenhouse grown plants, height was measured after 1 week of sowing and then on a 2-week basis using a total of eight replicates for each variety.

Several growth stages were collected in triplicate from plants grown in greenhouse according to the rice development system proposed by Counce et al. (2000) as detailed in Table 1. Collected samples were immediately frozen in N_2(l)_ and stored at -80°C. Petri dish germinated plants were used to collect the coleoptile at S3 which occurred approximately after 1 week of germination. For tissue sampling, tissue was collected at five-leaves plant stages (V5) and at flag leaf/panicle exertion (R2 and R3-R4) (Table 1). Finally, we collected the panicles at 50% of booting which occurs between R3 and R4 reproductive growth stages.

**Table 1:** Growth stages, according to the rice development system proposed by Counce et al. (2000), used for sample collection of the plants grown in the greenhouse.

Growth stage	Tissue	Code
S3 (emergence of prophyll from coleoptile)	Coleoptile	COL
V5 (collar formation on leaf 5 on main stem)	4th node	4N
	Internode between 4th and 5th node	4N5
	5th node	5N
	Basal part of the 5th leaf	B5L
	Apical part of the 5th leaf	A5L
R2 (flag leaf collar formation)	Node previous to the flag leaf node	pN
	Internode between flag leaf and previous leaf	pNF
	Flag leaf node	FN
	Basal part of the flag leaf	BFL
	Apical part of the flag leaf	AFL
R3-R4 (50% heading)	Panicle and florets	50H


### Chemicals and Material

All reagents were obtained from LabBox (Vilassar de Dalt, Spain). All phytohormones, unlabeled (GA_1_, GA_3_, GA_4_, GA_7_, GA_8_, GA_12_, GA_15_, GA_19_, GA_20_, GA_29_, GA_44_, GA_51_, GA_53_, ABA, JA, IAA) and deuterium-labeled (*d*_2_-GA_1_, *d*_2_-GA_3_, *d*_2_-GA_4_, *d*_2_-GA_7_, *d*_2_-GA_8_, *d*_2_-GA_12_, *d*_2_-GA_15_, *d*_2_-GA_19_, *d*_2_-GA_20_, *d*_2-_GA_29_, *d*_2_-GA_44_, *d*_2_-GA_51_, *d*_2_-GA_53_, *d*_6_-ABA, *d*_6_-JA, *d*_5-_IAA) standards, were purchased from OlChemIm (Olomouc, Czechia). SPE columns and OASIS^^®^^ HLB 1cc and OASIS^^®^^ PRIME HLB 1cc were purchased from Waters (Milford, MA, United States). Fixed insert vials and pre-slit PTFE screw cap were purchased from Teknokroma (Sant Cugat del Vallès, Spain). The HPLC column Kinetex^^®^^ 2.6 μm XB-C18 100 Å (30^∗^2.1 mm) was purchased from Phenomenex (Torrance, CA, United States), and the HPLC column Mediterranea Sea 18 column (10^∗^0.2 cm, 2.2 μm) was purchased from Teknokroma (Sant Cugat del Vallès, Spain).

### HPLC-MS Analysis

First of all, gibberellins were identified in rice samples using an HPLC-HRMS method, named HPLC-1 which is described in Table 2. Positive identification of phytohormones was based on the accurate mass measurement with an error of two mDa using high-resolution LTQ Orbitrap Velos mass spectrometer. An inventory of 16 phytohormones (14 gibberellins, ABA, JA, and IAA) was defined. Their theoretical exact masses were determined using a spectrum simulation tool of Xcalibur. Then, a list of possible candidates fitting the specific exact mass was generated using formula determination tools (elemental composition search) of Thermo Fischer Scientific Xcalibur softwares. The elemental number for phytohormones was restricted to include C, H, and O. The formula constraints for gibberellins were 19≤C≥20, 22≤H≥28H, 4≤O≥7, whereas for ABA, JA, and IAA the restriction was the exact formula for each compound. The search was based on single mass analysis and only considered the *m/z*-value of the monoisotopic peak. Considering temptative identified phytohormones, we proceed to buy them and to inject in the HPLC-1 system. In this way, we confirmed the presence of 13 gibberellins as well as ABA, JA, and IAA in rice samples.

**Table 2:** HPLC and MS conditions and parameters for HPLC-1 and HPLC-2 methods.

Conditions		HPLC-1	HPLC-2
**HPLC**			
	LC system	Agilent 1290 Infinity LC System (Santa Clara, CA, United States)	Accela chromatograph (Thermo Scientific, Hemel Hempstead, United Kingdom)
	Column	Kinetex^^®^^ 2.6 μm XB-C18 100 Å (30^∗^2.1 mm) (Phenomenex, Torrance, CA, United States)	Mediterranea Sea 18 column (10^∗^0.21 cm, 2.2 μm) (Teknokroma, Sant Cugat del Vallès, Spain)
	Column T	30°C	30°C
	Injection volume	10 μL	10 μL
	Flow rate	400 μL min-1	600 μL min-1
	Mobile phase	A: 0.05% of HAc; B: methanol	A: 0.05% of HAc; B: methanol
	Gradient elution (t, %B)	0, 20 → 5, 40 → 20, 90 → 30, 90 → 32, 20 → 40, 20	0, 20 → 2, 50 → 10, 90 → 13, 90 → 13.10, 20 → 15, 20
**MS**			
	MS System	LTQ Orbitrap Velos mass spectrometer (Thermo Scientific, Hemel Hempstead, United Kingdom)	6500 QTRAP^^®^^ MS/MS System (AB Sciex, Framingham, MA, United States)
	Ionization mode	ESI (-)	IonDrive (-)
	Resolution	30,000 at *m/z* 400	Unit
	Acquisition	FTMS: *m/z* 100 to 1000	Scheduled MRM (Multiple Reaction Monitoring)
	Operation parameters^∗^	Source voltage: -3.5 kV, sheath gas: 50 au, auxiliary gas: 20 au, sweep gas: 20 au, capillary temperature: 375°C	Ionspray voltage: -4.5 kV, nebulizer gas: 50 au, auxiliary gas: 40 au, curtain gas: 30 au, collision gas: high (au), focusing potential: -200 V, entrance potential: -10 V, declustering potential (DP) and collision energy (CE) can be revised in Table 3.


*^∗^au, arbitrary units.*

Once phytohormones were identified and the SPE protocol optimized, phytohormones from the rice samples collected and listed in Table 1 were extracted using three independent replicates, using an internal deuterium-labeled standard for each phytohormone, with the method C’ (explained below) and quantified in an HPLC-QqQ instrument with a method called HPLC-2 described in Table 2. Multiple reaction monitoring (MRM) mode was used to identify and quantify analytes. MS/MS parameters for working in MRM mode were optimized by direct infusion of each individual standard at a concentration of 0.1 mg ⋅ L^-1^ in MeOH:H_2_O (20:80, v/v) with 0.05% of HAc into the mass spectrometer using a syringe pump (Harvard Apparatus, Holliston, MA) at a constant flow rate of 10 μL ⋅ min^-1^. The scheduled MRM mode was employed instead of conventional MRM, which allows the simultaneous monitoring of multiple transitions by using retention time windows. To establish these windows, individual standard solutions were injected into the HPLC-MS/MS system to find their retention times, and RT windows were then estimated based on peak widths. Analyst 1.6.2 Software was used for data acquisition and MultiQuant 3.0.1 for data processing both from ABSciex (Framingham, MA, United States).

### Phytohormone SPE (Solid Phase Extraction) Protocols Test

**FIGURE 1:**
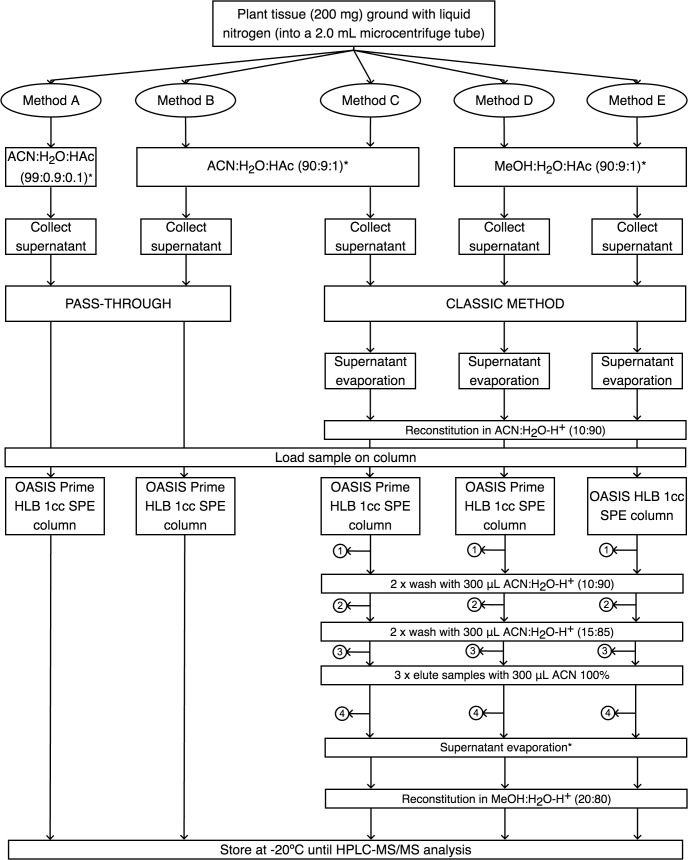
Flow chart of the phytohormone extraction protocols. In each numbered bullet, the fraction (#1, #2, #3) and eluate (#4) were collected to detect for possible phytohormone leakage. ACN, acetonitrile; HAc, acetic acid; MeOH, methanol; SPE, solid phase extraction; ^∗^, supplemented with 0.05% HAc.

A total of five methods were tested that consisted in combinations of extraction solutions, extraction methods and SPE columns. The three different extraction media (ACN: acetonitrile, HAc, acetic acid, MeOH: methanol) used were as follows: (i) ACN:H_2_O:HAc (99:0.9:0.1, v/v/v; as suggested by the manufacturer), (ii) ACN:H_2_O:HAc (90:9:1, v/v/v), and (iii) MeOH:H_2_O:HAc (90:9:1, v/v/v) (Figure 1). The two different extraction methods were: (i) pass-through and (ii) classic (Figure 1). For the classical method, we evaporated the sample in an Eppendorf concentrator 5301 (Hamburg, Germany) during 20 min for complete evaporation of the solvent, reconstituted in 200 μL of ACN:H_2_O (10:90, v/v) with 0.05% of HAc and then loaded on its corresponding column. For the pass-through approach we tested two methods: A and B using OASIS^^®^^ PRIME HLB columns, whereas for classical approach three methods were tested: C, D, and E we used OASIS^^®^^ PRIME HLB and OASIS^^®^^ HLB. Each method’s flow chart can be observed in Figure 1.

For the five methods, four independent replicates of 200 mg of frozen NRVC980385 leaves were grounded to a fine powder in N_2(l)_ using a pistil and a mortar. The ground tissue was mixed in a relation 1:4 with its corresponding extraction medium for each method (see Figure 1). To each sample, a pool of standard containing five GAs (GA_1_, GA_3_, GA_4_, GA_12_, GA_53_) and *d_2_*-GA_3_ at 5 μg ⋅ mL^-1^ of each one was added to the mortar. The resulting solution is transferred to a microcentrifuge tube and centrifuged during 12 min at 14000 *g*. The resulting supernatant is transferred to a new microcentrifuge tube and the remaining pellet is re-extracted with a 1/4 of the extraction medium volume added previously. This pellet is centrifuged during 12 min at 14000 *g* and the supernatant is transferred to the microcentrifuge tube containing the first supernatant.

For methods A and B, sample was directly loaded in the Oasis Prime HLB 1cc SPE columns using the pass-through approach and the eluate was collected on a fixed insert vial with a screw cap and stored at -20°C until analysis. For methods C, D and E, samples were evaporated and reconstituted in ACN:H_2_O (10:90, v/v) with 0.05% of HAc. In method D, the Oasis HLB 1cc SPE column was conditioned with MeOH and water, whereas for methods C and E sample was directly loaded onto the Oasis Prime HLB 1cc SPE columns. For methods C, D and E, all washing fractions (#1, #2, and #3) and the eluate (#4) from the column were collected to determine if phytohormones were lost in any step (Figure 1).

All samples obtained using the five methods (A, B, C, D, and E) were analyzed by the HPLC-1 method explained above (Table 2). To determine the effectiveness of each protocol tested, the deuterated gibberellin *d_2_*-GA_3_ at 5 μg ⋅ mL^-1^ each one was run with each sample. Afterward, peak area of *d_2_*-GA_3_ was compared with a standard directly loaded on a vial with a screw cap that was injected in the same way as the samples to calculate the recovery percentage of *d_2_*-GA_3_. After comparing the five protocols, a slightly modified method C was established and tested using six independent NRVC980385 leaf samples. In this new method, named C’, the evaporated samples were reconstituted in 600 μL ACN:H_2_O (5:95, v/v) with 0.05% of HAc and loaded in the Oasis Prime HLB 1cc SPE column. Sample was washed twice with 300 μL ACN:H_2_O (5:95, v/v) with 0.05% of HAc and eluted three times with 300 μL ACN 100% which was collected in a microcentrifuge tube. This solution was evaporated and the sample reconstituted in 200 μL MeOH:H_2_O (20:80, v/v) with 0.05% of HAc, and then transferred on a fixed insert vial with a screw cap and stored at -20°C until analysis.

### Statistical Analysis

Height data in the three varieties was verified for normality and homoscedasticity for each week of measurement. It was observed that all data showed normal distribution except for weeks 17, 19, and 21 when using the Shapiro–Wilkinson test and an α = 0.05. Each week data was heteroscedastic using the Levene’s test for homoscedasticity except for data of the first week (W1). For homoscedastic data, a one-way ANOVA test, which is very robust and accept transgressions to normality, followed by a Tukey *post hoc* test were used. On the other hand, for heteroscedastic data, a Kruskal–Wallis test for non-parametric data followed by a Conover-Iman *post hoc* test were used. For all tests, differences were considered to be significant at a probability of 5% (*p* < 0.05).

For the five extraction protocols tested (A, B, C, D, and E) and method C’, a one-way ANOVA followed by a Tukey *post hoc* test was performed on the fraction with the highest recovery percentages, after checking that the data was homoscedastic and normal using Levene’s and Shapiro–Wilkinson tests, respectively.

Phytohormone content (reported as ng ⋅ g^-1^ of fresh weight) was normalized for life cycle stages S3 and V5, and stages R2 and R3-R4 separately using the formula: x’ = (x_i_-x_min_)/(x_max_-x_min_). For representing all the data, heatmaps were used using the normalized data for each couple of life cycle stages. In addition, for each phytohormone, normality and homoscedasticity were checked for the three varieties in each tissue (COL, 4N, 4N5, 5N, B5L, A5L, pN, pNF, FN, BFL, AFL, 50H) using Shapiro–Wilkinson and Levene’s tests, respectively. Normal and homoscedastic data was analyzed using a one-way ANOVA followed by a Tukey *post hoc* test, normal and heteroscedastic data was analyzed using Kruskal–Wallis followed by a Conover-Iman multiple non-parametric pairwise test, and not normal and heteroscedastic data was analyzed using Welch’s ANOVA followed by a Games-Howell *post hoc* test. Supplementary Table S1 shows the statistical *p-*values and *F*-values for each of the analysis performed on each tissue and phytohormone.

## Results

### Mediterranean japonica Rice Varieties Characterization

**FIGURE 2:**
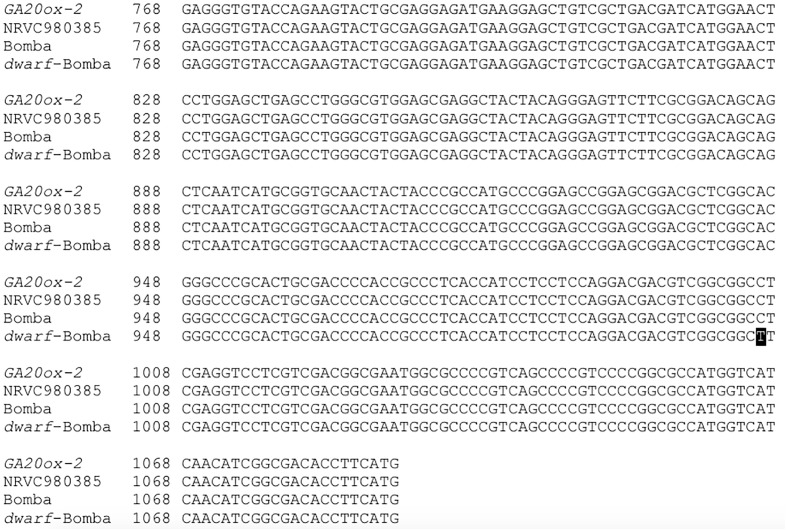
Alignment of the second exon of *GA20ox-2*, with the corresponding base numbers. The Calrose mutation present in *dwarf*-Bomba is blackened.

**FIGURE 3:**
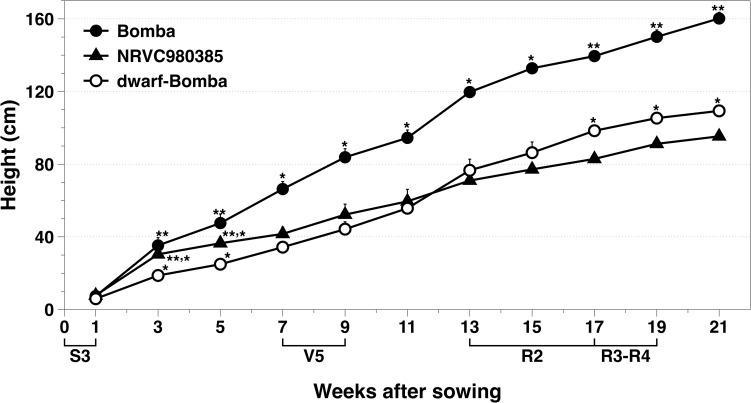
Monitoring of the rice heights of the three Mediterranean japonica rice varieties used. Values correspond to Asterisks indicate significant differences between rice varieties in each measurement week.

The phenotypical dwarfism trait of *dwarf*-Bomba observed in the field was checked by genotypic analysis. With Sanger sequencing we verified that it had a mutation, that corresponded to the Calrose mutation. Figure 2 shows the substitution of G by A in the position 1006 of the second exon, which corresponds to the Calrose mutation. The development of the three Mediterranean japonica rice varieties (NRVC980385, Bomba and *dwarf*-Bomba) was registered through height measurement (Figure 3). It can be seen that Bomba is significantly taller than *dwarf*-Bomba and NRVC980385 starting from week three and seven, respectively. The trend of Bomba being taller than the other two varieties is constant throughout the measurement period. Moreover, it is worth noting that between weeks eleven and thirteen the height of *dwarf*-Bomba increases rapidly, making this variety significantly taller than NRVC980385 from week 17 although having the *GA20ox* gene mutated.

### Phytohormone Extraction Protocol and HPLC-MS/MS Optimization

**FIGURE 4:**
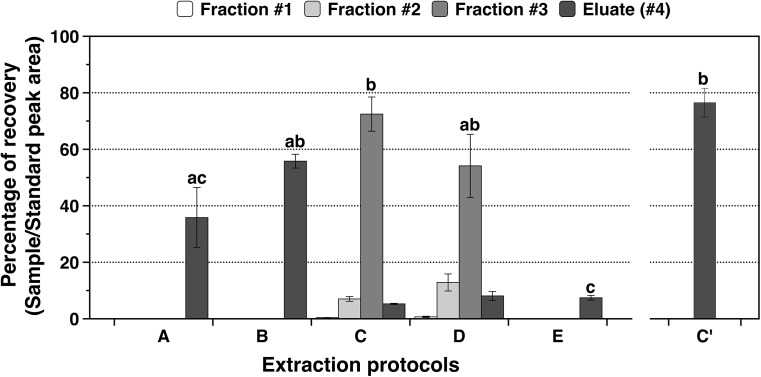
Recovery percentages for all the fractions (#1, #2, and #3) and eluates (#4) of the five extractions protocols (A, B, C, D, and E) and new method (C’) on NRVC980385 leaves. Methods detail for A to E can be seen on Figure 1. Columns correspond to mean ± SEM of 4 replicates for methods A to E whereas mean ± SEM of 6 replicates for method C’, letters above bars indicate significant differences between varieties for each tissue analyzed (Tukey tests at *p* < 0.05).

Five methods for extracting phytohormones (i.e., methods A, B, C, D, and E) were tested in this study (Figure 1). All methods display a good peak of the hormone *d_2_*-GA_3_ in the trace chromatogram (Supplementary Figure S1). The washing fractions as well as the eluate of all the methods were analyzed to determine the best solution to clean the columns before elution (Figure 4). When comparing the highest recovery percentage of each method (including C’), significant differences were observed between them (ANOVA: *F* = 13.96; *p-*value < 0.0001). Methods E displayed the significantly lower recovery percentage in any of the elutes, followed closely by method A (Figure 4). It can be observed for methods C and D that the eluate (#4) is low compared to the fraction #3 elute, because the second wash [ACN:H_2_O (15:85) with 0.05% of HAc] dragged the majority of *d_2_*-GA_3_ out of the column. In fact, the recovery percentage for those two methods is very good in the fraction #3 (67.2 ± 12.0 and 41.0 ± 6.4, mean ± SEM, respectively). On the other hand, method B although faster than C and D has lower recovery percentage if all fractions (#1, #2, and #3) and eluate (#4) of method C and D are, respectively summed together. The low recovery percentages in fractions is probably due to the fact that sample contains an excessive percentage of water. For method C, the recovery percentage is 85.1 ± 7.1 (mean ± SEM) for the sum of all fractions and eluate. Therefore, method C was chosen but some adjustments were performed in order to not lose phytohormones during the washes, and this method was established as C’. Washes in method C’ are performed with a ACN:H_2_O (5:95) solution with 0.05% of HAc, which corresponds to the fraction #1 that displayed a phytohormone recovery of 0.4%, and the elution was performed with 100% ACN. The recovery percentage was 76.4 ± 5.0 (mean ± SEM) corroborating the validity of this new method C’ (Figure 4).

Furthermore, method HPLC-1 was further optimized into method HPLC-2 in which a full phytohormone can be carried out in 20 min of chromatography instead of in 40 min as observed in Supplementary Figure S2. In Supplementary Figure S2, it can be observed that the retention time (RT) of *d_2_*-GA_8_ is similar for both methods, and in contrast the RT of *d_2_*-GA_12_ is almost half the time for HPLC-2 compared to HPLC-1. Moreover, detecting the gibberellins in the QTRAP6500 (QqQ; HPLC-2) instead of the LTQ Orbitrap Velos (HRMS; HPLC-1) allows to have higher sensibility. In fact, the signal to noise ratio (S/N) was 5 and 1.5 times higher in HPLC-2 than in HPLC-1 for *d_2_*-GA_8_ and *d_2_*-GA_12_, respectively (Supplementary Figure S2).

The declustering potential and collision energy parameters of the MS and MS/MS were optimized to generate the highest signal intensities for each phytohormone (Table 3 and Supplementary Figure S2). A scheduled MRM method was established with an MRM detection window of 60 s and a target scan time of 0.7 s. In Supplementary Figure S3, trace chromatograms of the 13 detected gibberellins in a sample (a replicate of *dwarf*-Bomba FN) can be observed showing a clear peak for each phytohormone. Quantification of gibberellins was done by the isotopic dilution method. Calibration curve was constructed with standard solutions between 0.2 and 200 ng ⋅ mL^-1^ diluted in MeOH:H_2_O (20:80 v/v) with 0.05% of HAc. The linear range for each hormone is presented in Table 3 and for the majority it displays a broad linear range that goes between 0.2 and 200 ng ⋅ mL^-1^. Linear regression was adjusted (1/x or 1/x^2^) in order to have accuracies between 80 and 120% for all the standards. Moreover, limit of detection (LOD) and limit of quantification (LOQ) was calculated for each phytohormone (gibberellins, ABA, JA, and IAA) as the concentration of phytohormone in a phytohormone extract derived from a rice sample that gives a S/N = 3 for LOD and S/N = 10 for LOQ (Table 3). LODs are very low for the majority of phytohormones, ranging from 0.1 to 1.6 pg ⋅ g^-1^ (f.w.) Good reproducibility was observed, as the relative standard deviations (RSDs) for a standard pool varied between 0.3 and 1.6%. High RSD was found for IAA and GA_44_ (20.0 and 9.3%, respectively) when standards of 0.4 ppb were analyzed, as this concentration is near the LOD values for those phytohormones.

**Table 3:** Multiple reaction monitoring transitions, retention time (RT), declustering potential (DP), collision energy (CE), limit of detection (LOD) and limit of quantification (LOQ) for the phytohormones analyzed in method HPLC-2 (6500QTRAP).

Compound	MRM transition	RT (min)	DP (V)	CE (V)	LOD^a^ (pg ⋅ g^-1^)	LOQ^a^ (pg ⋅ g^-1^)	Linear range (ng ⋅ mL^-1^)	RSD (%, 0.4 ng ⋅ mL^-1^)^b^	RSD (%, 40 ng ⋅ mL^-1^)^b^
GA_1_	347.0/259.0	2.6	-65	-26	0.4	1.2	0.2–200	1.9	0.9
*d_2_*-GA_1_	349.0/261.2	2.6	-140	-26	–	–	–	–	–
GA_3_	345.0/239.1	2.5	-95	-20	0.2	0.5	0.2–175	1.3	0.5
*d_2_*-GA_3_	347.0/241.1	2.5	-105	-20	–	–	–	–	–
GA_4_	331.0/243.2	5.6	-105	-26	0.7	2.2	0.2–200	5.4	0.7
*d_2_*-GA_4_	333.0/259.0	5.6	-120	-32	–	–	–	–	–
GA_7_	329.0/223.1	5.3	-80	-26	0.3	0.8	0.2–175	1	0.4
*d_2_*-GA_7_	331.0/225.1	5.3	-115	-24	–	–	–	–	–
GA_8_	363.0/275.2	1.7	-105	-24	0.7	2.3	0.2–90	1.5	0.4
*d_2_*-GA_8_	364.9/277.0	1.7	-125	-24	–	–	–	–	–
GA_12_	331.0/313.1	8.4	-20	-38	0.4	1.2	0.2–175	2.5	0.3
*d_2_*-GA_12_	333.0/315.2	8.4	-145	-36	–	–	–	–	–
GA_15_	329.0/257.0	6.7	-20	-34	0.1	0.3	0.2–90	0.5	0.3
*d_2_*-GA_15_	331.0/259.1	6.7	-35	-34	–	–	–	–	–
GA_19_	361.1/273.0	4.6	-60	-34	0.1	0.4	0.2–200	2.5	0.7
*d_2_*-GA_19_	362.9/275.0	4.6	-115	-36	–	–	–	–	–
GA_20_	331.1/225.2	3.9	-105	-34	0.5	1.8	0.2–200	2.4	0.7
*d_2_*-GA_20_	332.9/227.1	3.9	-130	-34	–	–	–	–	–
GA_29_	347.1/259.1	1.9	-120	-24	0.4	1.2	0.2–90	0.9	0.9
*d_2_*-GA_29_	348.9/261.2	1.9	-135	-22	–	–	–	–	–
GA_44_	345.1/272.9	4.3	-130	-34	0.2	0.6	0.2–175	9.3	0.9
*d_2_*-GA_44_	347.0/275.0	4.3	-120	-36	–	–	–	–	–
GA_51_	331.1/243.1	4.7	-105	-22	0.5	1.6	0.2–200	2.1	0.4
*d_2_*-GA_51_	332.9/245.0	4.7	-105	-24	–	–	–	–	–
GA_53_	347.1/189.0	6.0	-125	-46	0.2	0.5	0.2–200	7.1	0.3
*d_2_*-GA_53_	349.0/188.9	6.0	-120	-48	–	–	–	–	–
ABA	263.1/153.1	3.4	-55	-16	0.1	0.3	0.2–200	1.1	0.8
*d_6_*-ABA	269.0/159.0	3.4	-95	-16	–	–	–	–	–
JA	209.1/58.9	4.1	-70	-16	0.1	0.4	0.2–200	1.2	0.9
*d_6_*-JA	215.0/62.2	4.1	-60	-18	–	–	–	–	–
IAA	174.0/129.9	2.7	-95	-16	1.6	5.2	0.2–200	20.0	1.6
*d_5_*-IAA	178.9/135.0	2.7	-35	-16	–	–	–	–	–


*^*a*^Limits of detection (LOD) and quantification (LOQ) were determined in rice samples and are expressed as pg ⋅ g^-*1*^ of fresh weight. ^*b*^Values are average of ten replicates.*

### Gibberellin Profiling of Three Mediterranean japonica Rice Varieties

**FIGURE 5:**
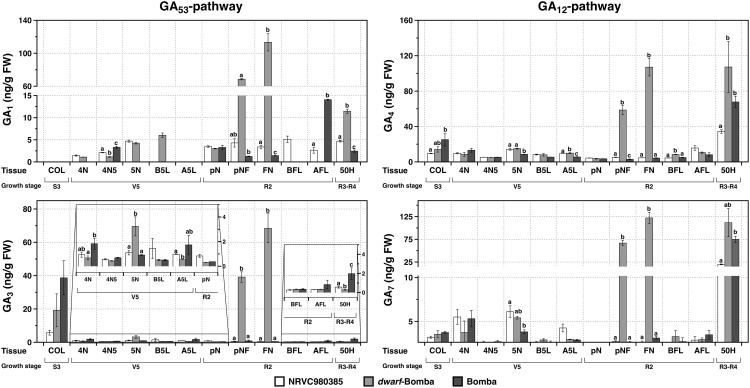
Content of the bioactive gibberellins (GA_1_, GA_3_, GA_4_, and GA_7_) in the different tissues for the three analyzed varieties: NRVC980385, *dwarf-*bomba, and Bomba. COL, coleoptile; 4N, 4th node; 4N5, internode between 4th and 5th node; 5N, 5th node; B5L, basal part of the 5th leaf; A5L, apical part of the 5th leaf; pN, node previous to the flag leaf node; pNF, internode between flag leaf and previous leaf; FN, flag leaf node; BFL, basal part of the flag leaf; AFL, apical part of the flag leaf. Columns correspond to mean ± SEM of 3 replicates, and letters above bars indicate significant differences between varieties for each tissue analyzed.

Regarding the bioactive gibberellins, it is worth noting that their contents in the different tissues is different being GA_4_ in average the one displaying the highest values (Figure 5) for the three varieties. In addition, GA_1_ is not detected in the coleoptile (COL) whereas the other three bioactive gibberellins are present, and have a similar pattern in which Bomba is the one displaying the highest values followed by *dwarf*-bomba and then NRVC980385. Moreover, this same gibberellin, GA_1_, in contrast with the other three bioactive gibberellins (GA_3_, GA_4_, and GA_7_) was not detected in all varieties in the tissue A5L, and also not detected in several of the other analyzed tissues. Moreover, depending on the tissue, the phytohormone contents greatly varies, such is the case of COL where the contents of GA_3_, GA_4_, and GA_7_ are higher in Bomba compared to the other two varieties (NRVC980385 and *dwarf*-Bomba). On one hand, regarding the tissue 4N, bioactive gibberellin contents are always higher in Bomba than *dwarf*-Bomba. On the other hand, when the tissue 5N is looked thoroughly, the opposite is observed where the content of all the four bioactive gibberellins is always significantly higher in *dwarf*-Bomba than in Bomba. Interestingly, GA_7_ was almost not detected in the tissue 4N5 and completely absent in the tissue pN. Contrary, GA_3_ and GA_4_ were quantified in all the tissues of the three analyzed varieties. Strikingly, the content of the four bioactive gibberellins is significantly highly increased in *dwarf*-bomba for the internode between flag leaf and previous leaf (pNF) and the flag leaf node (FN) compared to the other two varieties (Figure 5 and Supplementary Table S1). Similarly, the content of the bioactive gibberellins in the panicle and florets (50H) is higher in *dwarf*-Bomba compared to Bomba (although concentrations are lower), and significantly higher for both when compared to NRVC980385 (Figure 5 and Supplementary Table S1).

**FIGURE 6:**
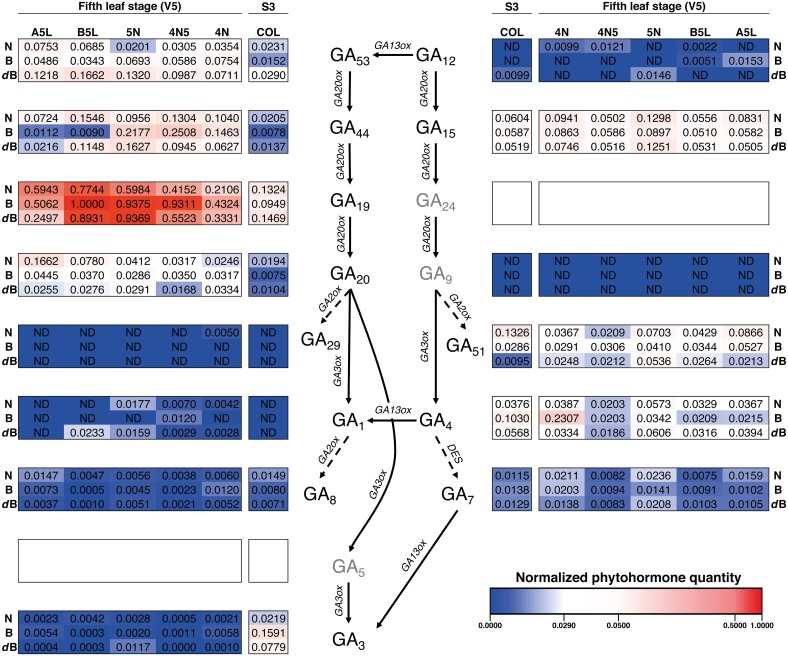
Heatmap of the hormone profiling of the three Mediterranean japonica rice varieties in the stages S3 and V5. Normalized phytohormone quantity scale is shown at the lower right of the image and corresponds to the mean of 3 replicates. Three phytohormones (GA_5_, GA_9_, and GA_24_, in gray) were not analyzed, ND, not determined. N, NRVC980385; B, Bomba; *d*B, *dwarf*-bomba; COL, coleoptile; 4N, 4th node; 4N5, internode between 4th and 5th node; 5N, 5th node; B5L, basal part of the 5th leaf; A5L, apical part of the 5th leaf.

It seems that phytohormones GA_19_, of the GA_53_-pathway, is a key intermediate for the normal development of rice plants, as its levels are very high in all varieties for Stages S3 and V5 (Figure 6 and Supplementary Figure S4). More in detail, the first phytohormone profiling was performed for the life cycle stage S3 (emergence of prophyll from coleoptile) where it is noteworthy that GA_29_ and GA_1_, both of the GA_53_-pathway, were not detected in neither of the three rice varieties (Figure 6 and Supplementary Figure S5). In this same gibberellin pathway, only GA_3_ showed high values which were higher in Bomba. Concomitantly, GA_4_ of the GA_12_-pathway also showed high values for Bomba as well as other phytohormones, without one GA with an elevated concentration, which contrasts to the tendency observed for the gibberellins of the GA_53_-pathway.

When focusing on the five-leaves plants (Stage V5), GA_1_ was barely detected in other tissues, similar to what was observed in COL. Similarly, GA_29_ was only detected for NRVC980385 in the 4th node (4N, Supplementary Figure S5). On one hand, the content of GA_44_ and GA_20_ for the majority of tissues analyzed in this growth stage for the three varieties were increased, with a concomitant increase of GA_19_, when compared to the coleoptile (COL). On the other hand, content levels of GA_12_, GA_15_, and GA_51_ were not very different to those obtained in COL, being GA_12_ even not detected in several tissues (Supplementary Figure S6). Finally, it is worth noting that GA_15_ contents are very similar between all varieties and tissues. In general, for both growth stages, S3 (coleoptile) and V5 (fifth-leaf stage), it seems that the GA_53_-pathway could serve as a pool for GAs as it intermediates have higher concentrations and their bioactive GAs (GA_1_ and GA_3_) are present at low levels, in contrast both bioactive GAs of the GA_12_-pathway, GA_4_ and GA_7_, have higher concentrations than GA_1_ and GA_3_ (Figure 6).

**FIGURE 7:**
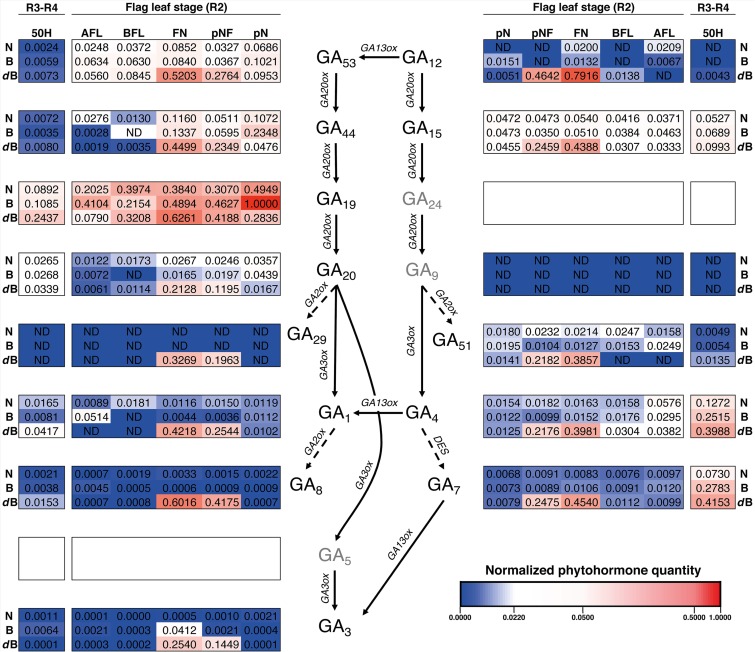
Heatmap of the hormone profiling of the three Mediterranean japonica rice varieties in the stages R2 and R3-R4. Normalized phytohormone quantity scale is shown at the lower right of the image and corresponds to the mean of 3 replicates. Three phytohormones (GA_5_, GA_9_, and GA_24_, in gray) were not analyzed, ND, not determined phytohormone. N, NRVC980385; B, Bomba; *d*B, *dwarf*-bomba; pN, node previous to the flag leaf node; pNF, internode between flag leaf and previous leaf; FN, flag leaf node; BFL, basal part of the flag leaf; AFL, apical part of the flag leaf.

Regarding growth stages R2 and R3-R4, GA_19_ also appears to have a key role as its contents is the highest of all the analyzed (Figure 7 and Supplementary Figure S4). As in stages S3 and V5, GA_29_ was almost undetected in all tissues and GA_1_ was not detected in three tissues. As observed in S3 and V5, GA_15_ contents were all similar between tissues and varieties for R2 and R3-34 (Figure 7 and Supplementary Figure S6). Interestingly, all the gibberellin contents, in both the GA_53_- and the GA_12_-pathway, are increased in the pNF and FN for *dwarf*-Bomba compared to the other two varieties. Moreover, when looking at R3-R4 for the same variety, where Panicle and florets (50H) show a higher content (Figure 7 and Supplementary Figures S4, S5). Finally, for tissues R2 and R3-R4 we observe the same trend as in tissues S3 and V5, where GA_53_-pathway has a higher accumulation of intermediates GAs when compared with the GA_12_-pathway.

### ABA, JA, and IAA Profiling of Three Mediterranean japonica Rice Varieties

In addition to the 13 gibberellins detected and quantified in several tissues and growth stages of rice tissues, our method allows to analyze in addition the phytohormones abscisic acid (ABA) and jasmonic acid (JA) in all tissues and varieties; indole-3-acetic acid (IAA) (Supplementary Figure S7). ABA concentrations were in general very similar among the three varieties, except for the coleoptile (COL) where *dwarf*-Bomba has the higher values and both parts of the leaf in the V5 growth stage where Bomba has the higher values. JA levels were very similar in all tissues and varieties displaying significant differences in only two tissues (pNF in R2 and 50H in R3-R4, Supplementary Table S1). In addition, it is also noteworthy that IAA in the flag leaf (stage R2) was absent in almost all the varieties. Finally, in the heading stage (50H) no IAA was detected in Bomba whereas NRVC980385 and *dwarf*-Bomba displayed very high values.

## Discussion

It is well established that studying phytohormones in plants is crucial for understanding several developmental and physiological processes, including tolerance to different stresses. We have established a protocol for analyzing and quantifying more than 15 phytohormones, including a total of 13 different gibberellins, in different rice tissues with detection at trace levels. To our knowledge, this is the first study that has reported such a high number of analyzed gibberellins at the same time in rice (*Oryza sativa* ssp. japonica). We have also established that bioactive gibberellins content in rice tissues not only depends on the presence of a wild-type GA20 oxidase 2 gene (*GA20ox-2*), but it must also depend on whether or not other bioactive GAs are present in the variety that contains the Calrose mutation. Interestingly, we found that (i) dwarf plants do not have a drastically lower gibberellin content in comparison to their non-dwarf counterpart and (ii) their growth is primarily halted only in the early stages as its growth is faster in the later phenological stages.

In this work, a reliable and broad phytohormone extraction protocol for rice was developed. Acetonitrile was a better organic solvent than methanol as the recovery percentages were highest in methods A, B, and C. This is corroborated by other studies (Flores et al., 2011; Urbanová et al., 2013; Cui et al., 2015). Urbanová et al. (2013) also reported that acetonitrile extract less interfering pigments than methanol. It is crucial to achieve high recovery percentages, because a loss of phytohormones during extraction could lead to wrong detection and, therefore, to results misinterpretation (Chen et al., 2012; Cui et al., 2015). The best acetonitrile method was C, and it was further improved into method C’, and tested in leaf samples which yielded recovery percentages similar to other broad profiling protocols (Chen et al., 2012; Urbanová et al., 2013). Moreover, our method is simpler than others, because only one SPE columns is used, whereas normally others authors employ two or even three columns for sample purification (Kojima et al., 2009; Chen et al., 2012; Urbanová et al., 2013). In addition, the relative standard deviation we found in our samples is very low when compared to the study made by Chen et al. (2012), suggesting that our method is very precise. The only exceptions were IAA and GA_44_ when analyzed at a 0.4 ppb concentration, but this is due to the fact that this concentration is within the LOD values for those phytohormones. In addition, the linear range at which standards were measured is broad, ranging from 0.2 to 200 ng ⋅ mL^-1^ for several phytohormones, which allows our method to be used in a wide range of sample concentrations. Finally, our method shows good detectability (e.g., 0.1 pg ⋅ g^-1^ (f.w.) for four phytohormones), good reproducibility (no more than 1.6 at 40 ppb for standards) and good separation of all the studied gibberellins as it does not have interferences between isobaric species (e.g., between GA_4_ and GA_51_ or *d_2_*-GA_7_ and GA_20_).

In this work a broad phytohormone profiling was performed, which allows to analyze changes during growth development. In fact, changes between different developmental stages and even tissues within a developmental stage were observed in the three varieties. The first notorious finding was that independently of the variety, GA_1_ was absent in the coleoptile which is in disagreement with an article published by Liu L. et al. (2018). Nevertheless, in that study GA content was measured after 4 days of germination whereas in our study measurement was done after 7 days, therefore this particular GA may not be needed for coleoptile elongation. In this same tissue, it was clear that GA_4_ and GA_3_ are the most important bioactive gibberellins, as their concentration is more than 15 times higher for Bomba. In agreement with this, it has been shown that low levels of GA_4_ in *Arabidopsis thaliana* are related to no germination of seeds, proving that GA_4_ is a crucial bioactive gibberellin in the coleoptile (Yamaguchi, 2008). Moreover, Kaneko et al. (2003) have shown that the embryo has differential gibberellin genes expression patterns which suggests that the genes for GA_3_ and GA_4_ could be under- and over-expressed, respectively. In fact, the low availability of GA_1_, final active products of the GA_53_-pathway, could be explained by the high quantities detected in their precursor gibberellins, specially GA_19_. In addition, GA_3_ detection and quantification in all of the tissues and species analyzed is in agreement with the studies by Ma et al. (2011) and Wu et al. (2012).

Concerning gibberellins production in different tissues, as also reported by Kojima et al. (2009), we evidenced higher bioactive GA levels in the nodes compared to the internodes in both V5 and R2 growth stages. These findings are supported by Kaneko et al. (2003), that showed higher activity of *OsGA3ox2* and *OsGA20ox2* in the node of elongating stems. In the later phenological stages, such as heading, GA contents have been shown to be high which is also in correlation with our findings (Yang et al., 2000). GA_7_ and GA_3_ levels throughout the development of the three varieties had in general low concentrations when compared with GA_4_, suggesting that the latter is the key active gibberellin in this species. This is in concordance with Binenbaum et al. (2018) that claim that GA_7_ and GA_3_ are biologically active but present at minor levels. Moreover, the high availability of GA_4_ is also related to the intermediate GAs concentrations, as in this pathways (GA_12_) the intermediates are present at low levels when compared with those of the GA_53_-pathway. The exception was *dwarf*-Bomba which showed high levels of GA_1_, GA_3_, GA_4_, and GA_7_ at pNF and FN, which could explain its faster growth in later phenological stages.

Regarding the other three phytohormones also studied in this work, it is worth noting that both JA and ABA are present throughout the plant development. The high levels observed in Bomba for B5L and A5L compared to the other two varieties are expected since this phytohormone has a crucial role in stomata movements (López-Carbonell et al., 2009). In contrast to JA and ABA, IAA is almost absolutely absent in Bomba at the R2 and R3-R4 stages, but has elevated levels in the heading stage (R3-R4) for NRVC980385 and *dwarf*-Bomba. Since IAA has been shown to be increased during heading (Yang et al., 2000), it is surprising that Bomba levels are so low compared to those of the three varieties have high GA levels. These results for JA, ABA, and IAA are not conclusive but give insights in phytohormone mechanisms in different tissues, therefore more studies are needed to fully understand hormone patterns during life cycles.

Sanger sequencing results confirmed that only *dwarf*-Bomba had a mutation in the *GA20ox-2* gene that corresponded to a deletion between exon 1 and exon 2. This deletion has been reported by other authors and is one of the four *GA20ox-2* mutations that lead to the semi-dwarf varieties (Ashikari et al., 2002; Monna et al., 2002; Sasaki et al., 2002; Spielmeyer et al., 2002; Hedden, 2003). The deletion clearly explains the semi-dwarfism A9m traits observed by other authors the 21 weeks of plant development: Bomba (traditional variety) was significantly higher than its mutated counterpart, *dwarf*-Bomba. Strangely, NRVC980385 was also significantly shorter than Bomba throughout the monitoring. The height values observed for NRVC980385 are in accordance with those of the literature [e.g., NRVC9830 in Serrat et al. (2014)], and those reported for Bomba are not surprising since traditional varieties are known to have higher heights (Franquet Bernis and Borràs Pàmies, 2004; Okuno et al., 2014). Therefore, heights differences observed are not exclusively caused by the well-studied *sd-1* mutation. Interestingly, NRVC980385 during the first half of rice development was higher than *dwarf*-Bomba even though its *GA20ox-2* gene is not mutated.

In fact, it is known that other genes involved in GAs biosynthesis and signaling pathways as well as other phytohormones are also contributing to height in rice (Liu F. et al., 2018). JA and IAA levels were very similar in tissues of the V5 growth stage between NRVC980385 and *dwarf*-Bomba, so their difference in height might respond to changes in other GA genes or phytohormones such as brassinosteroids (BRs) or strigolactones (SLs) (Liu F. et al., 2018). Surprisingly, during week 11 and 13, *dwarf*-Bomba height surpasses that of NRVC980385 which is in correlation with the elevated contents of bioactive gibberellins of both the GA_53_- (GA_1_ and GA_3_) and the GA_12_-pathway (GA_4_ and GA_7_) reported in the pNF and the FN. This increment in GAs levels are most likely caused by either other one or all of the other three *GA20ox* genes [i.e., *GA20ox-1, GA20ox-3*, and *GA20ox-4*, Sakamoto (2004)]. As gibberellins are crucial for internode elongation, this is the phenotypical characteristic that should explain the height increment in *dwarf*-Bomba during flag leaf collar formation stage (Counce et al., 2000; Ayano et al., 2014; Wang et al., 2017).

## Conclusion

In conclusion, we have shown that GA_1_ is not a crucial gibberellin in the rice coleoptiles neither in more advanced phenological stages, because its levels are in general low. Moreover, GA_19_ seems to have a crucial role in gibberellin availability in rice as its levels were much higher than all the other gibberellins in all tissues. In addition, it has been demonstrated that the *GA20ox-2* mutation is not the only factor affecting height in rice, as a mutated variety had an increased growth during flag leaf collar formation stage (R2). It was corroborated that GA_3_ and GA_7_ are present at low levels in the majority of rice tissues. Finally, all these findings were possible due to the establishment, for the first time, of a simple and broad phytohormone extraction and detection protocol that allows to identify 13 gibberellins and ABA, JA, and IAA in several tissues at different phenological stages.

## Data availability

All datasets for this study are included in the manuscript and the Supplementary Files.

## Author Contributions

CL-C, ML-C, and SN were in charge of the study design. CL-C and XS were in charge of the implementation of greenhouse cultures. CL-C and OJ were in charge of the implementation of the HPLC-MS/MS method. ML-C and CL-C were in charge of the study interpretation and reporting. CL-C was in charge of the writing of the study.

## Conflict of Interest Statement

The authors declare that the research was conducted in the absence of any commercial or financial relationships that could be construed as a potential conflict of interest.

## References

[B1] Ashikari M. Sasaki A. Ueguchi-Tanaka M. Itoh H. Nishimura A. Datta S. (2002). Loss-of-function of a rice gibberellin biosynthetic gene, GA20 oxidase (GA20ox-2), led to the rice ‘Green Revolution’. *Breed. Sci.* 52 143–150. 10.1270/jsbbs.52.143

[B2] Ayano M. Kani T. Kojima M. Sakakibara H. Kitaoka T. Kuroha T. (2014). Gibberellin biosynthesis and signal transduction is essential for internode elongation in deepwater rice. *Plant Cell Environ.* 37 2313–2324. 10.1111/pce.12377 24891164 PMC4282320

[B3] Binenbaum J. Weinstain R. Shani E. (2018). Gibberellin localization and transport in plants. *Trends Plant Sci.* 23 410–421. 10.1016/j.tplants.2018.02.005 29530380

[B4] Chen M. L. Fu X. M. Liu J. Q. Ye T. T. Hou S. Y. Huang Y. Q. (2012). Highly sensitive and quantitative profiling of acidic phytohormones using derivatization approach coupled with nano-LC-ESI-Q-TOF-MS analysis. *J. Chromatogr. B Anal. Technol. Biomed. Life Sci.* 905 67–74. 10.1016/j.jchromb.2012.08.005 22917596

[B5] Counce P. A. Keisling T. C. Mitchell A. J. (2000). A uniform, objective, and adaptive system for expressing rice development. *Crop Sci.* 40 436–443. 10.2135/cropsci2000.402436x

[B6] Cui K. Lin Y. Zhou X. Li S. Liu H. Zeng F. (2015). Comparison of sample pretreatment methods for the determination of multiple phytohormones in plant samples by liquid chromatography-electrospray ionization-tandem mass spectrometry. *Microchem. J.* 121 25–31. 10.1016/j.microc.2015.02.004

[B7] Davies P. J. (ed.) (2010). *Plant Hormones: Biosynthesis, Signal Transduction, Action!*. 3rd Edn. New York, NY: Springer.

[B8] Doyle J. Doyle J. (1987). A rapid DNA isolation procedure for small quantities of fresh leaf tissue. *Phytochem. Bull.* 19 11–15.

[B9] Flores M. I. A. Romero-González R. Frenich A. G. Vidal J. L. M. (2011). QuEChERS-based extraction procedure for multifamily analysis of phytohormones in vegetables by UHPLC-MS/MS. *J. Sep. Sci.* 34 1517–1524. 10.1002/jssc.201100093 21595026

[B10] Franquet Bernis J. M. Borràs Pàmies C. (2004). *Variedades y Mejora del Arroz (Oryza sativa L.)*, 1st Edn. Tortosa: Dialnet Foundation.

[B11] Hedden P. (2001). Gibberellin metabolism and its regulation. *J. Plant Growth Regul.* 20 317–318. 10.1007/s003440010039 11986757

[B12] Hedden P. (2003). The genes of the green revolution. *Trends Genet.* 19 5–9. 12493241 10.1016/s0168-9525(02)00009-4

[B13] Hedden P. Phillips A. L. (2000). Gibberellin metabolism: new insights revealed by the genes. *Trends Plant Sci.* 5 523–530. 10.1016/S1360-1385(00)01790-8 11120474

[B14] Hedden P. Thomas S. G. (2012). Gibberellin biosynthesis and its regulation. *Biochem. J.* 444 11–25. 10.1042/BJ20120245 22533671

[B15] Iqbal N. Masood A. Khan N. A. (2012). “‘Phytohormones in sailinity tolerance: ethylene and gibberellins cross talk’,” in *Phytohormones and Abiotic Stress Tolerance in Plants*, eds Khan N. A. Nazar R. Iqbal N. Anjum N. A. (New York, NY: Springer), 77–98. 10.1007/978-3-642-25829-9

[B16] Kaneko M. Itoh H. Inukai Y. Sakamoto T. Ueguchi-Tanaka M. Ashikari M. (2003). Where do gibberellin biosynthesis and gibberellin signaling occur in rice plants? *Plant J.* 35 104–115. 10.1046/j.1365-313X.2003.01780.x 12834406

[B17] Kojima M. Kamada-Nobusada T. Komatsu H. Takei K. Kuroha T. Mizutani M. (2009). Highly sensitive and high-throughput analysis of plant hormones using ms-probe modification and liquid chromatographytandem mass spectrometry: an application for hormone profiling in *Oryza sativa*. *Plant Cell Physiol.* 50 1201–1214. 10.1093/pcp/pcp057 19369275 PMC2709547

[B18] Kudo T. Akiyama K. Kojima M. Makita N. Sakurai T. Sakakibara H. (2013). UniVIO: a multiple omics database with hormonome and transcriptome data from rice. *Plant Cell Physiol.* 54 1–12. 10.1093/pcp/pct003 23314752 PMC3583028

[B19] Liu F. Wang P. Zhang X. Li X. Yan X. Fu D. (2018). The genetic and molecular basis of crop height based on a rice model. *Planta* 247 1–26. 10.1007/s00425-017-2798-1 29110072

[B20] Liu L. Xia W. Li H. Zeng H. Wei B. Han S. (2018). Salinity inhibits rice seed germination by reducing α-amylase activity via decreased bioactive gibberellin content. *Front. Plant Sci.* 9:925. 10.3389/fpls.2018.00275 29556245 PMC5845124

[B21] López-Carbonell M. Gabasa M. Jáuregui O. (2009). Enhanced determination of abscisic acid (ABA) and abscisic acid glucose ester (ABA-GE) in *Cistus albidus* plants by liquid chromatography-mass spectrometry in tandem mode. *Plant Physiol. Biochem.* 47 256–261. 10.1016/j.plaphy.2008.12.016 19167901

[B22] Ma Q. Hedden P. Zhang Q. (2011). Heterosis in rice seedlings: its relationship to gibberellin content and expression of gibberellin metabolism and signaling genes. *Plant Physiol.* 156 1905–1920. 10.1104/pp.111.178046 21693671 PMC3149939

[B23] Macías J. M. Pournavab R. F. Reyes-Valdés M. H. Benavides-Mendoza A. (2014). Development of a rapid and efficient liquid chromatography method for determination of gibberellin A4 in plant tissue, with solid phase extraction for purification and quantification. *Am. J. Plant Sci.* 2014 573–583.

[B24] Monna L. Kitazawa N. Yoshino R. Suzuki J. Masuda H. Maehara Y. (2002). Positional cloning of rice semidwarfing gene, sd-1: rice ‘Green Revolution Gene’ encodes a mutant enzyme involved in gibberellin synthesis. *DNA Res.* 9 11–17. 11939564 10.1093/dnares/9.1.11

[B25] Okuno A. Hirano K. Asano K. Takase W. Masuda R. Morinaka Y. (2014). New approach to increasing rice lodging resistance and biomass yield through the use of high gibberellin producing varieties. *PLoS One* 9:e86870. 10.1371/journal.pone.0086870 24586255 PMC3929325

[B26] Olszewski N. Sun T.-P. Gubler F. (2002). Gibberellin signaling: biosynthesis, catabolism, and response pathways. *Plant Cell* 14 S61–S80. 10.1105/tpc.010476.GAs 12045270 PMC151248

[B27] Peleg Z. Blumwald E. (2011). Hormone balance and abiotic stress tolerance in crop plants. *Curr. Opin. Plant Biol.* 14 290–295. 10.1016/j.pbi.2011.02.001 21377404

[B28] Reddy I. N. B. L. Kim B. K. Yoon I. S. Kim K. H. Kwon T. R. (2017). Salt tolerance in rice: focus on mechanisms and approaches. *Rice Sci.* 24 123–144. 10.1016/j.rsci.2016.09.004

[B29] Sakamoto T. (2004). An overview of gibberellin metabolism enzyme genes and their related mutants in rice. *Plant Physiol.* 134 1642–1653. 10.1104/pp.103.033696 15075394 PMC419838

[B30] Sasaki A. Ashikari M. Ueguchi-Tanaka M. Itoh H. Nishimura A. Swapan D. (2002). Green revolution: a mutant gibberellin-synthesis gene in rice – new insight into the rice variant that helped to avert famine over thirty years ago. *Nature* 416 701–702. 10.1038/416701a 11961544

[B31] Serrat X. Cardona M. Gil J. Brito A. M. Moysset L. Nogués S. (2014). A mediterranean japonica rice (*Oryza sativa*) cultivar improvement through anther culture. *Euphytica* 195 31–44. 10.1007/s10681-013-0955-6

[B32] Spielmeyer W. Ellis M. H. Chandler P. M. (2002). Semidwarf (sd-1), ‘green revolution’ rice, contains a defective gibberellin 20-oxidase gene. *Proc. Natl. Acad. Sci. U.S.A.* 99 9043–9048. 10.1073/pnas.132266399 12077303 PMC124420

[B33] Urbanová T. Tarkowská D. Novák O. Hedden P. Strnad M. (2013). Analysis of gibberellins as free acids by ultra performance liquid chromatography-tandem mass spectrometry. *Talanta* 112 85–94. 10.1016/j.talanta.2013.03.068 23708542

[B34] Wang Y. Zhao J. Lu W. Deng D. (2017). Gibberellin in plant height control: old player, new story. *Plant Cell Rep.* 36 391–398. 10.1007/s00299-017-2104-5 28160061

[B35] Wu Q. Wu D. Duan C. Shen Z. Guan Y. (2012). Hollow fiber-based liquid-liquid-liquid micro-extraction with osmosis: II. Application to quantification of endogenous gibberellins in rice plant. *J. Chromatogr. A* 1265 17–23. 10.1016/j.chroma.2012.09.066 23089517

[B36] Yamaguchi S. (2008). Gibberellin metabolism and its regulation. *Annu. Rev. Plant Biol.* 59 225–251. 10.1007/s003440010039 18173378

[B37] Yang J. Peng S. Visperas R. M. Sanico A. L. Zhu Q. Gu S. (2000). Grain filling pattern and cytokinin content in the grains and roots of rice plants. *Plant Growth Regul.* 30 261–270. 10.1023/A:1006356125418

