# Supplementary material for: Phytohormone Profiling Method for Rice: Effects of GA20ox Mutation on the Gibberellin Content of Japonica Rice Varieties
Source: Front Plant Sci. 2019 Jun 7;10:733. doi: 10.3389/fpls.2019.00733 (PMC6565999; doi:10.3389/fpls.2019.00733)
Supplement: Supplementary file 1 [file Table_1.docx]

Supplementary Table S1. Statistical values (*p*-values and F) for the test used for analyzing each phytohormone in each tissue comparing the three varieties.

| **Growth stage** | **Tissue** | **GA_1_** | | **GA_3_** | | **GA_4_** | | **GA_7_** | | **GA_8_** | | **GA_12_** | |
| --- | --- | --- | --- | --- | --- | --- | --- | --- | --- | --- | --- | --- | --- |
|  |  | F | *p*-value | F | *p*-value | F | *p*-value | F | *p*-value | F | *p*-value | F | *p*-value |
| **S3** | **COL** | - | - | 4.02 | 0.07820^a^ | 2.81 | **0.21930^c^** | 1.12 | 0.38690^a^ | 6.47 | **0.03180^a^** | - | - |
| **V5** | **4N** | 7.56 | 0.05140^a^ | 4.11 | 0.07520^a^ | 0.61 | 0.60130^c^ | 0.85 | 0.47190^a^ | 6.18 | **0.03490^a^** | - | - |
|  | **4N5** | 59.50 | **0.00010^a^** | 11.67 | **0.00860^a^** | 3.38 | 0.10390^a^ | 0.57 | 0.59520^a^ | 1.44 | 0.30750^a^ | - | - |
|  | **5N** | 2.10 | 0.22110^a^ | 5.60 | **0.06080^b^** | 5.42 | 0.06650^b^ | 7.95 | **0.02060^a^** | 3.99 | 0.07900^a^ | - | - |
|  | **B5L** | - | - | 0.55 | 0.62140^c^ | 3.06 | 0.12160^a^ | 1.31 | 0.33620^a^ | 5.60 | **0.06080^b^** | 2.81 | 0.16890^a^ |
|  | **A5L** | - | - | 128.83 | **0.00080^c^** | 7.20 | **0.02730^b^** | 3.75 | 0.14910^c^ | 20.30 | **0.00210^a^** | - | - |
| **R2** | **pN** | 2.65 | 0.20940^c^ | 14.07 | **0.03620^c^** | 3.44 | 0.10130^a^ | 1.86 | 0.23500^a^ | 40.49 | **0.00030^a^** | 3.52 | **0.10620^c^** |
|  | **pNF** | 7.20 | **0.02730^b^** | 61.72 | **0.00450^c^** | 56.36 | **0.00260^c^** | 52.96 | **0.00240^c^** | 73.31 | **0.00140^c^** | - | - |
|  | **FN** | 58.63 | **0.00590^c^** | 24.63 | **0.01880^c^** | 43.00 | **0.00820^c^** | 41.38 | **0.00470^c^** | 30.12 | **0.01470^c^** | 83.65 | **0.00130^c^** |
|  | **BFL** | - | - | 1.14 | 0.38160^a^ | 5.96 | **0.05090^b^** | 0.36 | 0.83710^b^ | 4.36 | 0.11330^b^ | - | - |
|  | **AFL** | 3.86 | **0.04950^b^** | 2.11 | 0.25050^c^ | 3.58 | 0.09470^a^ | 0.75 | 0.51320^a^ | 5.42 | **0.06650^b^** | 3.41 | 0.13870^a^ |
| **R3-R4** | **50H** | 202.46 | **0.00000^a^** | 7.20 | **0.02730^b^** | 12.15 | **0.03890^c^** | 27.68 | **0.01570^c^** | 7.20 | **0.02730^b^** | - | - |
| a: ANOVA test; b: Kruskal-Wallis; c: Welch's ANOVA. | | | | | | | | | | | | | |

Table S1 continuation.

| **Growth stage** | **Tissue** | **GA_15_** | | **GA_19_** | | **GA_20_** | | **GA_29_** | | **GA_44_** | | **GA_51_** | | **GA_53_** | |
| --- | --- | --- | --- | --- | --- | --- | --- | --- | --- | --- | --- | --- | --- | --- | --- |
|  |  | F | *p*-value | F | *p*-value | F | *p*-value | F | *p*-value | F | *p*-value | F | *p*-value | F | *p*-value |
| **S3** | **COL** | 22.49 | **0.01370^c^** | 7.94 | **0.02810^a^** | 9.52 | **0.01380^a^** | - | - | 16.21 | 0.00380^a^ | 66.71 | 0.00350^c^ | 43.41 | 0.00030^a^ |
| **V5** | **4N** | 0.37 | 0.70460^a^ | 10.04 | **0.03630^c^** | 27.16 | **0.00750^c^** | - | - | 2.00 | 0.21660^a^ | 0.45 | 0.66020^a^ | 1.38 | 0.32050^a^ |
|  | **4N5** | 1.74 | 0.25380^a^ | 6.22 | **0.03440^a^** | 38.02 | **0.00040^a^** | - | - | 1.32 | 0.33380^a^ | 13.37 | 0.00610^a^ | 7.16 | 0.02570^a^ |
|  | **5N** | 7.09 | **0.02630^a^** | 0.69 | 0.53770^a^ | 0.78 | 0.50140^a^ | - | - | 3.47 | 0.09960^a^ | 23.69 | 0.00140^a^ | 4.54 | 0.06290^a^ |
|  | **B5L** | 0.94 | 0.44280^a^ | 0.93 | 0.44520^a^ | 6.88 | 0.08260^c^ | - | - | 2.68 | 0.23020^c^ | 5.76 | 0.09260^c^ | 18.57 | 0.00270^a^ |
|  | **A5L** | 6.51 | 0.08080^c^ | 0.94 | 0.44270^a^ | 1.10 | 0.43000^c^ | - | - | 3.82 | 0.14790^b^ | 10.37 | 0.01130^a^ | 1.98 | 0.21800^a^ |
| **R2** | **pN** | 0.10 | 0.91020^a^ | 7.20 | **0.02730^b^** | 8.33 | **0.01860^a^** | - | - | 10.19 | 0.05240^c^ | 1.27 | 0.40870^c^ | 0.77 | 0.50240^a^ |
|  | **pNF** | 51.84 | **0.00230^c^** | 0.69 | 0.53780^b^ | 5.60 | **0.06080^b^** | - | - | 14.49 | 0.00500^a^ | 58.85 | 0.00440^c^ | 5.42 | 0.06650^a^ |
|  | **FN** | 30.63 | **0.00880^c^** | 5.40 | **0.04550^a^** | 34.15 | **0.01200^c^** | - | - | 5.60 | 0.06080^b^ | 35.37 | 0.00510^c^ | 5.42 | 0.06650^a^ |
|  | **BFL** | 1.14 | 0.38020ª | 2.50 | 0.16200^a^ | 3.82 | 0.12240^a^ | - | - | 2.69 | 0.17600^a^ | 2.51 | 0.18850^a^ | 7.70 | 0.02200^a^ |
|  | **AFL** | 1.80 | 0.24390^a^ | 6.49 | **0.03900^a^** | 12.88 | **0.00670^a^** | - | - | 3.19 | 0.17940^c^ | 52.51 | 0.00190_a_ | 5.67 | 0.04140^a^ |
| **R3-R4** | **50H** | 37.40 | **0.00040^a^** | 19.18 | **0.00250^a^** | 0.66 | 0.55120^a^ | - | - | 8.51 | 0.01770^a^ | 4.14 | 0.14100^c^ | 13.39 | 0.01860^c^ |
| a: ANOVA test; b: Kruskal-Wallis test; c: Welch's ANOVA. | | | | | | | | | | | | | | | |

Table S1 continuation bis.

| **Growth stage** | **ABA** | | **JA** | | **IAA** | |
| --- | --- | --- | --- | --- | --- | --- |
|  | F | *p*-value | F | *p*-value | F | *p*-value |
| **S3** | 3.20 | 0.20190^b^ | 3.20 | 0.11350^a^ | 0.76 | 0.50720^a^ |
| **V5** | 0.33 | 0.73350^a^ | 0.08 | 0.92270^a^ | 2.76 | 0.25210^b^ |
|  | 1.31 | 0.39070^c^ | 0.16 | 0.86060^c^ | 1.38 | 0.32070^a^ |
|  | 0.07 | 0.93370^a^ | 1.87 | 0.39320^b^ | 4.38 | 0.06720^a^ |
|  | 15.16 | 0.00450^a^ | 1.42 | 0.49110^b^ | 0.17 | 0.84700^c^ |
|  | 9.28 | 0.01460^a^ | 3.20 | 0.11310^a^ | 0.12 | 0.88480^a^ |
| **R2** | 0.29 | 0.75610^a^ | 1.68 | 0.26340^a^ | 2.99 | 0.12570^a^ |
|  | 3.07 | 0.12100^a^ | 6.15 | 0.03530^a^ | 4.74 | 0.09510^a^ |
|  | 13.53 | 0.00600^a^ | 0.27 | 0.77500^a^ | 9.34 | 0.03780^a^ |
|  | 10.35 | 0.01140^a^ | 0.80 | 0.67030^a^ | - | - |
|  | 1.96 | 0.22070^a^ | 0.02 | 0.97620^a^ | - | - |
| **R3-R4** | 4.20 | 0.12710^c^ | 10.09 | 0.01200^a^ | 1.92 | 0.23770^a^ |
| a: ANOVA test; b: Kruskal-Wallis test; c: Welch's ANOVA. | | | | | | |
