# Supplementary figures and images for: Phytohormone Profiling Method for Rice: Effects of GA20ox Mutation on the Gibberellin Content of Japonica Rice Varieties
Source: Front Plant Sci. 2019 Jun 7;10:733. doi: 10.3389/fpls.2019.00733 (PMC6565999; doi:10.3389/fpls.2019.00733)

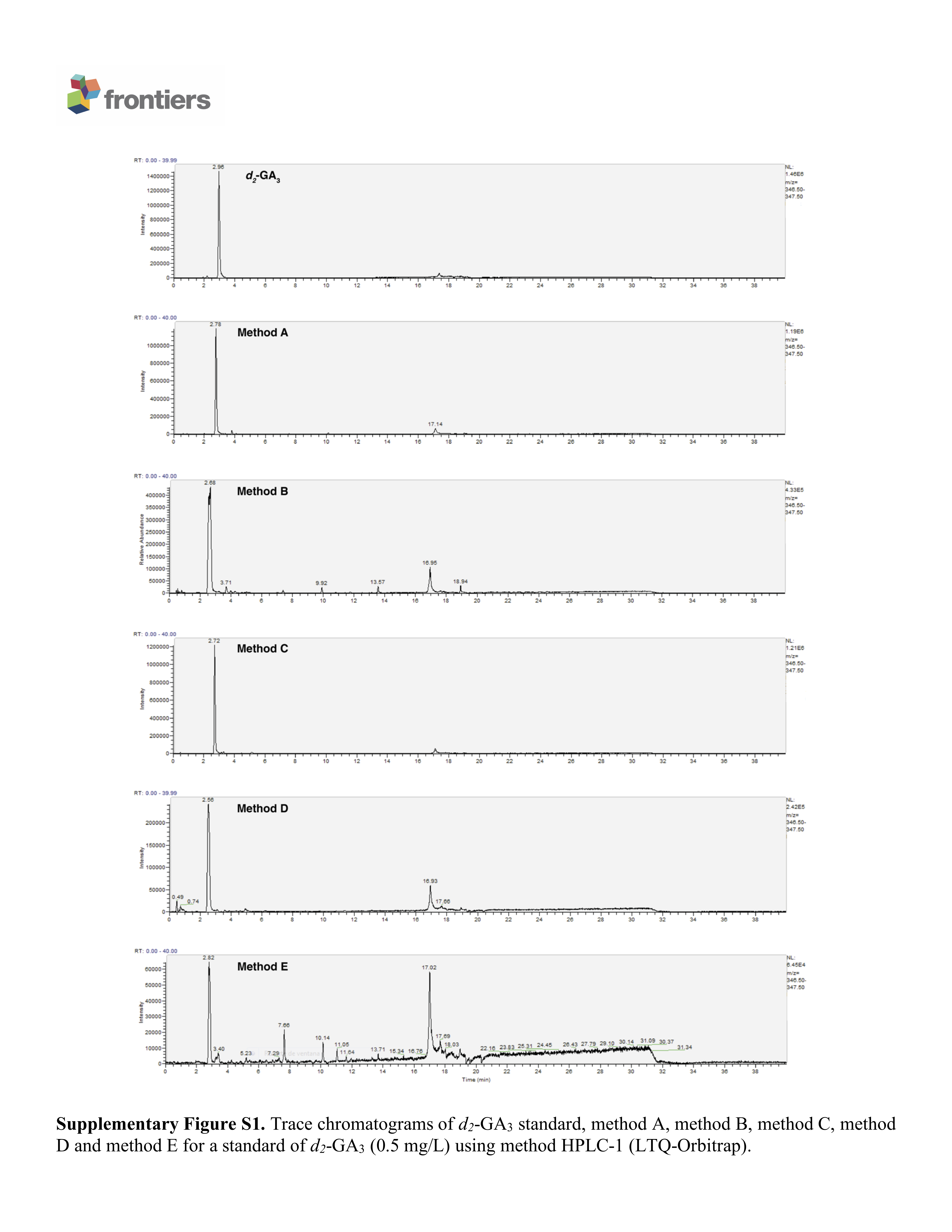

Supplement: Supplementary file 2 [file Image_1.png]

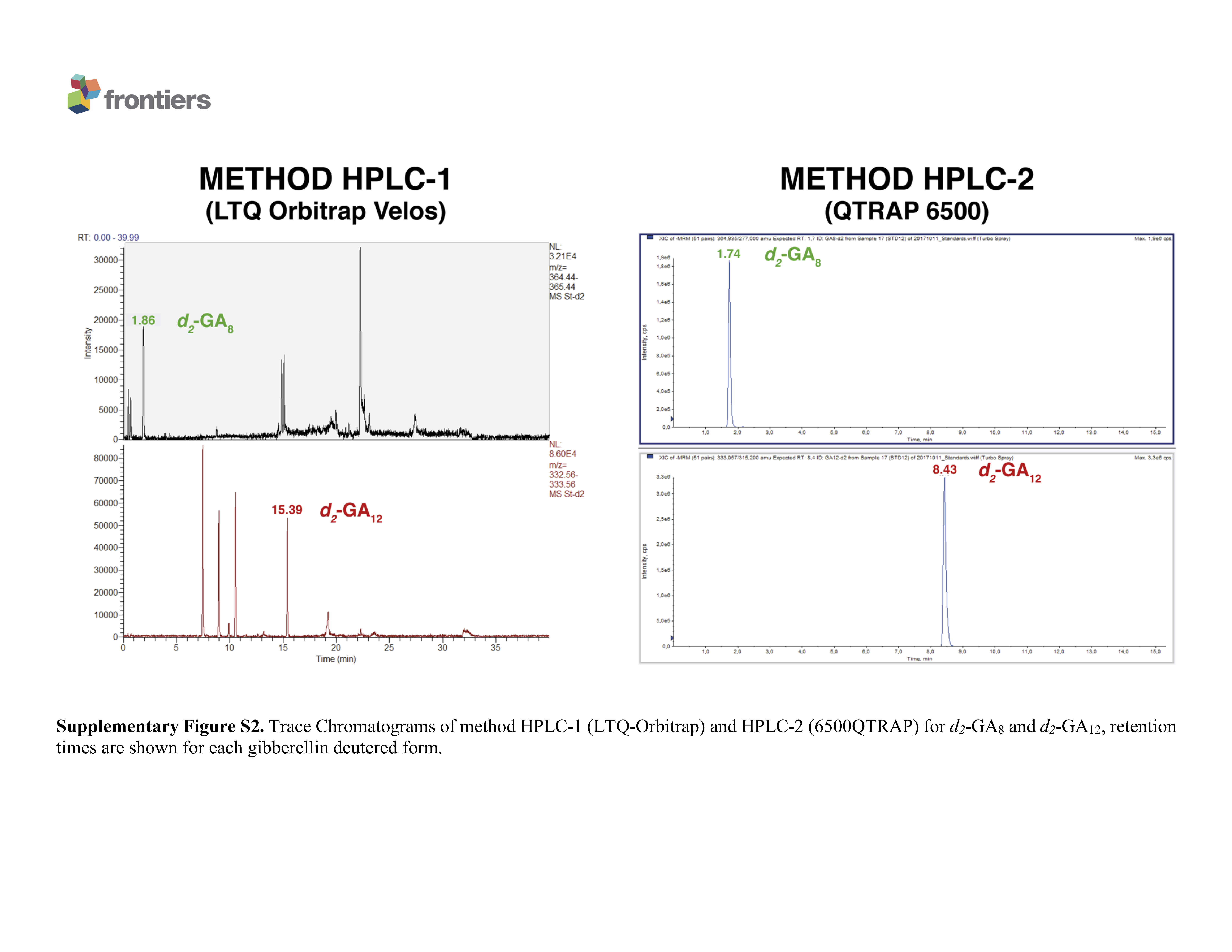

Supplement: Supplementary file 3 [file Image_2.png]

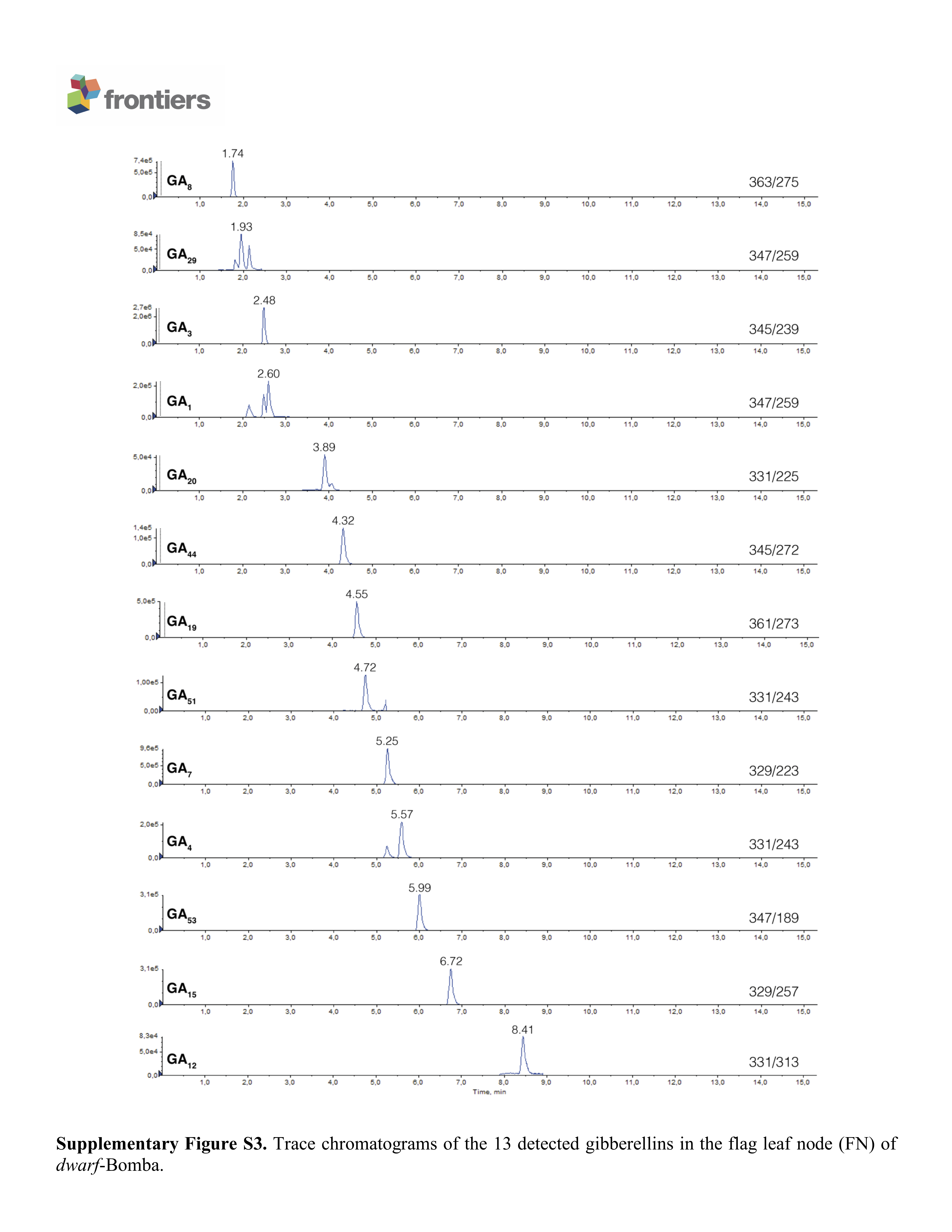

Supplement: Supplementary file 4 [file Image_3.png]

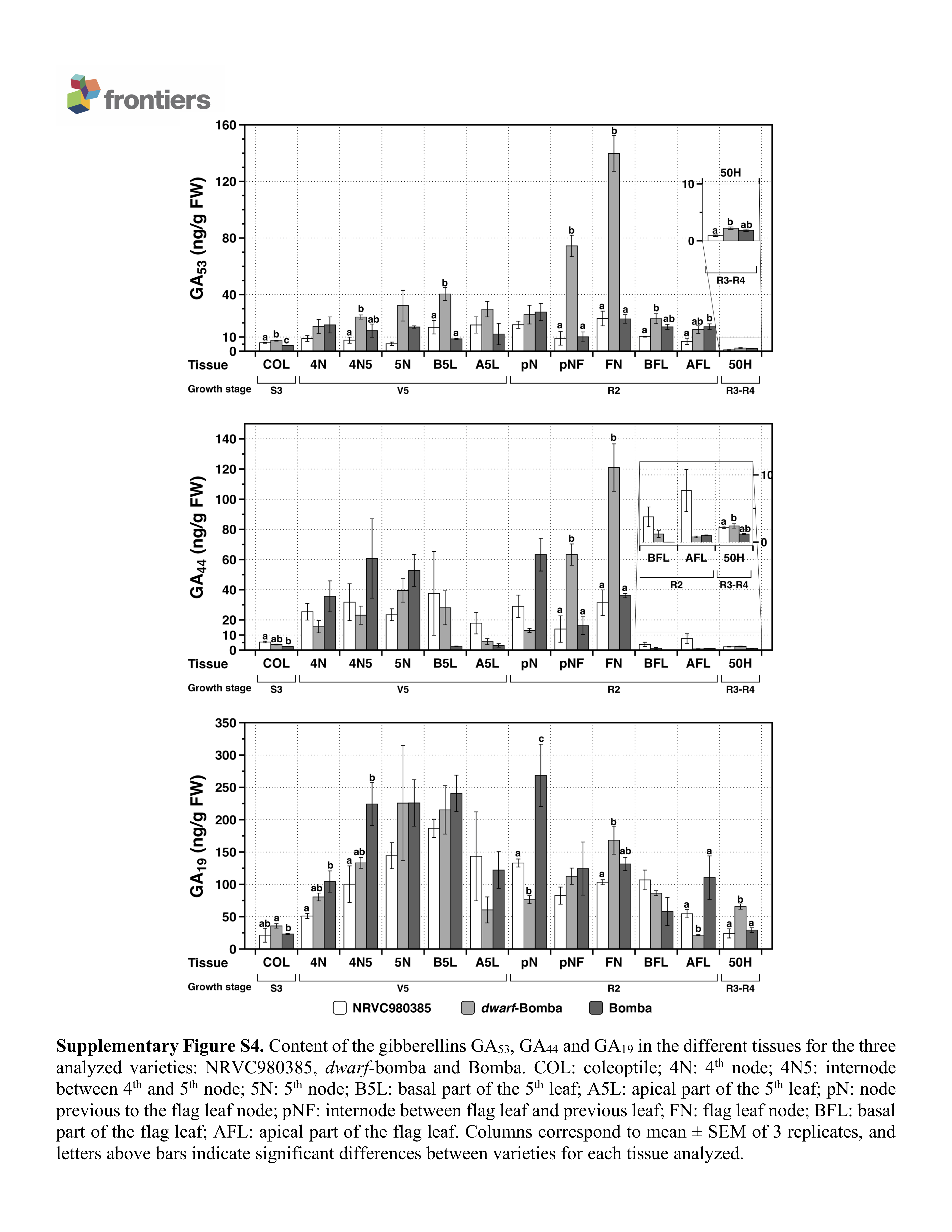

Supplement: Supplementary file 5 [file Image_4.png]

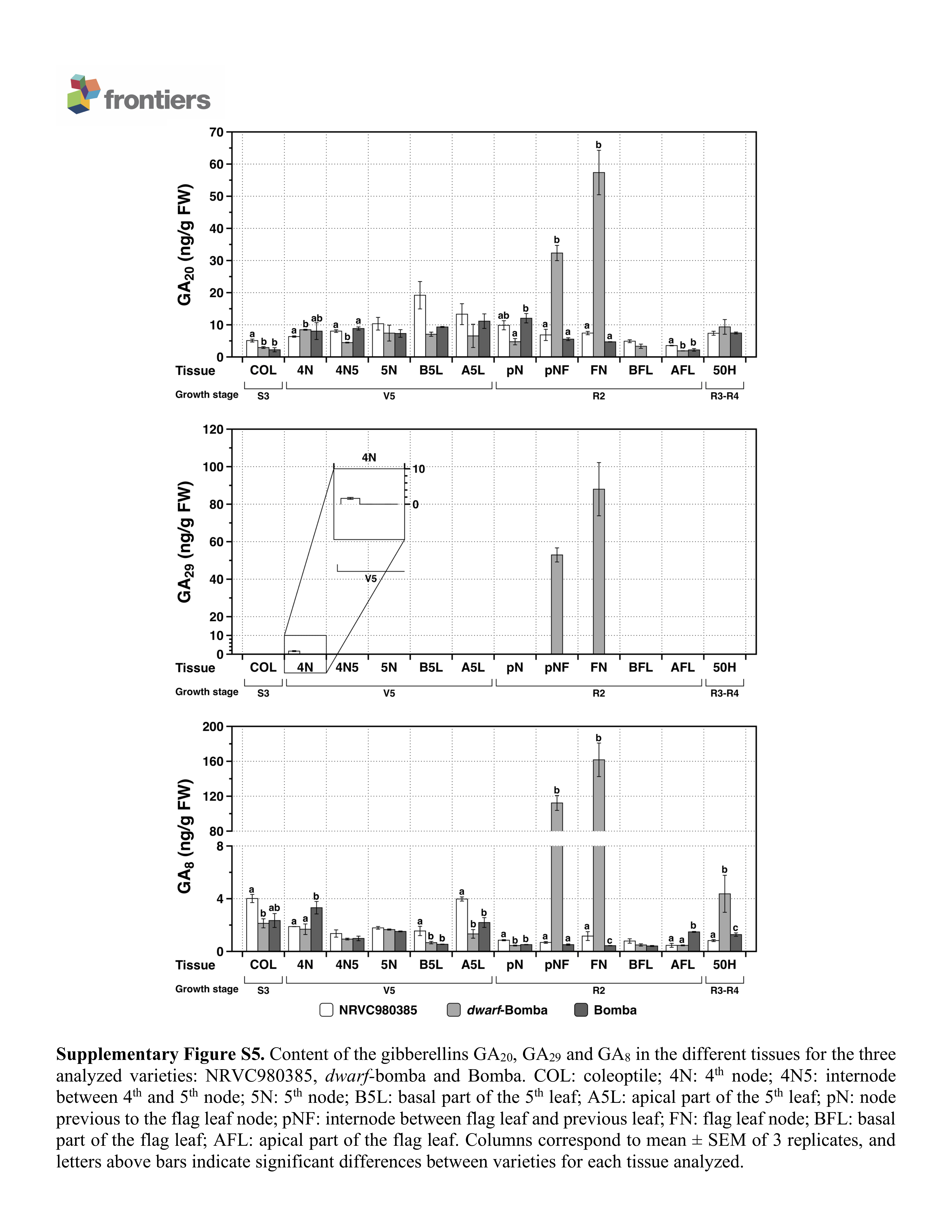

Supplement: Supplementary file 6 [file Image_5.png]

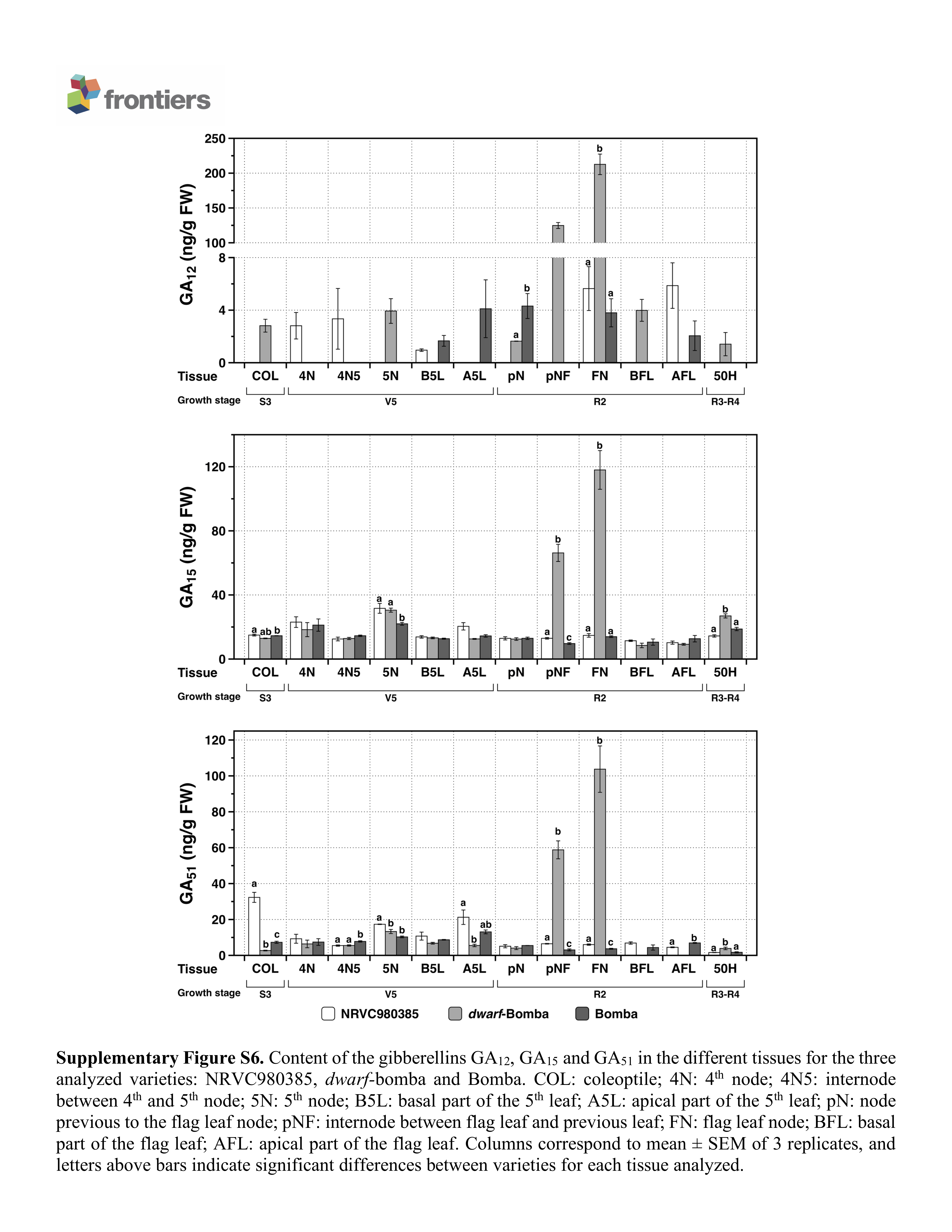

Supplement: Supplementary file 7 [file Image_6.png]

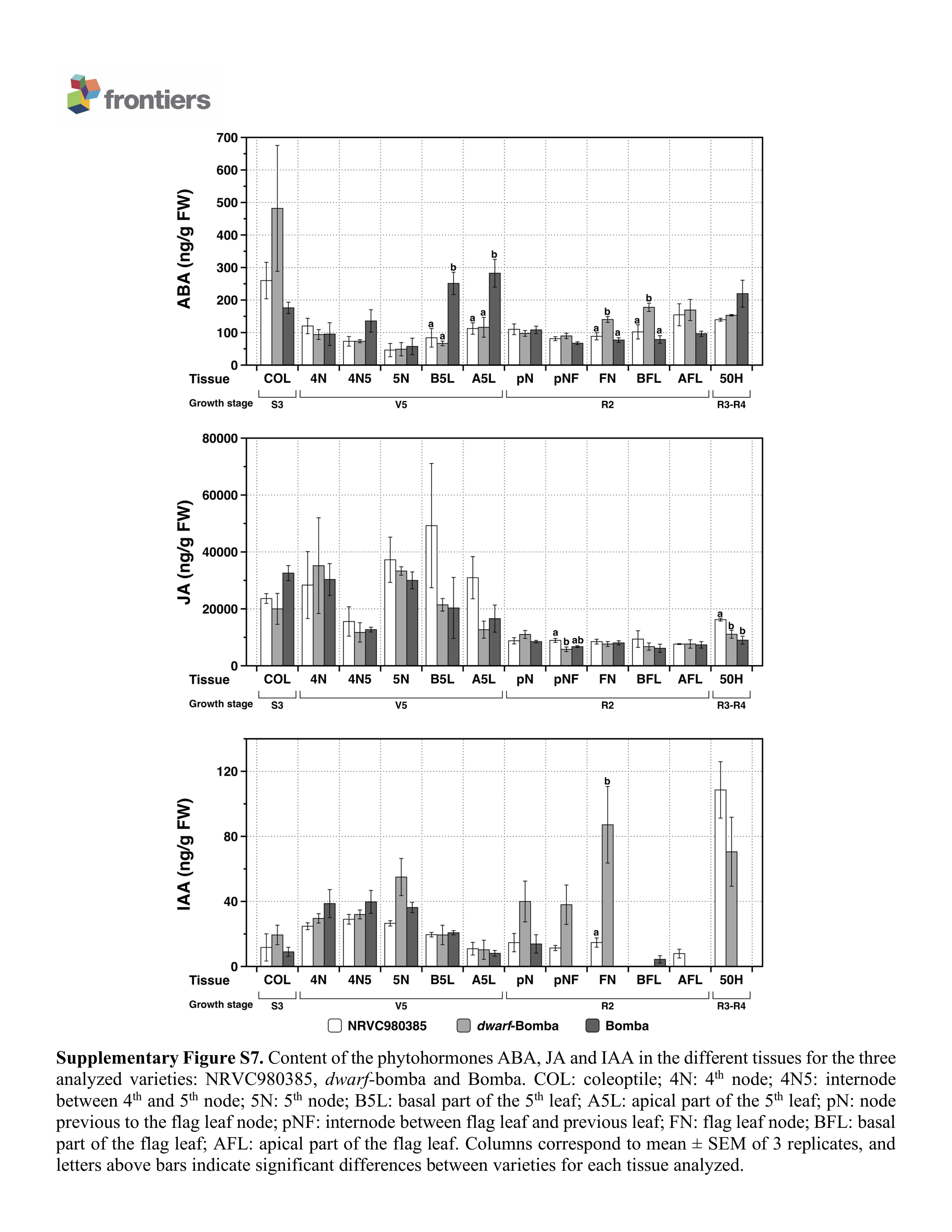

Supplement: Supplementary file 8 [file Image_7.png]
